# International guidelines for the *in vivo* assessment of skin properties in non-clinical settings: part 1. pH

**DOI:** 10.1111/srt.12016

**Published:** 2012-12-26

**Authors:** Aleksandr B Stefaniak, Johan du Plessis, Swen M John, Fritz Eloff, Tove Agner, Tzu-Chieh Chou, Rosemary Nixon, Markus F C Steiner, Irena Kudla, D Linn Holness

**Affiliations:** 1 National Institute for Occupational Safety and HealthMorgantown, WV, USA; 2 School for Physiology, Nutrition and Consumer Sciences, North-West UniversityPotchefstroom, South Africa; 3 Department of Dermatology, Environmental Medicine, Health Theory, University of OsnabrueckOsnabrueck, Germany; 4 Bispebjerg Hospital, University of CopenhagenCopenhagen, Denmark; 5 Department of Public Health, College of Public Health, China Medical UniversityTaichung, Taiwan; 6 Occupational Dermatology Research and Education Centre, Skin and Cancer Foundation IncCarlton, Vic., Australia; 7 Environmental and Occupational Medicine, University of AberdeenAberdeen, Scotland; 8 Dalla Lana School of Public Health, University of Toronto and Department of Occupational and Environmental Health, St. Michael's HospitalToronto, ON, Canada

**Keywords:** skin surface pH, workplace measurement, occupational skin diseases, irritation, allergy, contact dermatitis, skin barrier, skin absorption, stratum corneum, sweat

## Abstract

**Background:**

Skin surface pH is known to influence the dissolution and partitioning of chemicals and may influence exposures that lead to skin diseases. Non-clinical environments (e.g. workplaces) are highly variable, thereby presenting unique measurement challenges that are not typically encountered in clinical settings. Hence, guidelines are needed for consistent measurement of skin surface pH in environments that are difficult to control.

**Methods:**

An expert workshop was convened at the 5th International Conference on Occupational and Environmental Exposure of Skin to Chemicals to review available data on factors that could influence the determination of skin surface pH in non-clinical settings with emphasis on the workplace as a worst case scenario.

**Results:**

The key elements of the guidelines are: (i) minimize, to the extent feasible, the influences of relevant endogenous (anatomical position, skin health, time of day), exogenous (hand washing, barrier creams, soaps and detergents, occlusion), environmental (seasonality), and measurement (atmospheric conditions) factors; (ii) report pH measurements results as a difference or percent change (not absolute values) using a measure of central tendency and variability; and (iii) report notable deviations from these guidelines and other relevant factors that may influence measurements.

**Conclusion:**

Guidelines on the measurement and reporting of skin surface pH in non-clinical settings should promote consistency in data reporting, facilitate inter-comparison of study results, and aid in understanding and preventing occupational skin diseases.

Human skin is composed of three layers of stratified tissue (from outermost to innermost): stratum corneum (SC), viable epidermis, and the dermis. The SC is a hydrophobic layer of protein-rich stacked keratinocytes joined tightly together by intercellular lipids (1–3). The outer surface of the SC is coated with a co-mixture of aqueous sweat (electrolytes, amino acids, nitrogenous substances, etc.) and oily sebum lipids with minor amounts of intercellular lipid from the SC. The pH of the liquid coating on the outer surface of the SC is the product of both endogenous (i.e. phospholipid-free fatty acid pathway, sodium-proton transporters, and possibly the histidine-urocanic acid pathway) and exogenous (i.e. sweat and sebum secretions and their degradation products) factors (4). Among its functions, skin surface pH helps to maintain SC integrity and cohesion, regulate epidermal barrier homeostasis, and maintain microbial flora balance.

The skin surface pH may be altered depending upon disease status of the skin (5–8). Changes in skin surface pH can influence dissolution and/or partitioning of chemical contaminants that come into contact with the SC in the workplace. For example, *in vitro* dissolution of mild steel, chromium (VI), and gold often increases as artificial sweat pH increases (9–11). In contrast, dissolution of sensitizers, such as nickel and beryllium, increases as artificial sweat pH decreases (12–16). Skin diseases, such as allergic and irritant dermatitis, are a major economic burden for employers and employees alike (17, 18). In the United States, the economic burden of dermatitis exceeded one billion dollars in the year 2004 (18) and in Europe the economic burden is estimated greater than one billion Euro annually (19). Allergic dermatitis is of particular concern in the workplace because once a person is sensitized, only lifelong avoidance of repeat exposure can prevent elicitation of a reaction. Development of allergic and irritant dermatitis places a significant burden on workers who may experience pain and itching, scaling of skin, embarrassment from their skin condition, interference with work, and/or delays in returning to work or having to change jobs to avoid exposure often resulting in a loss of income (17, 20, 21). Employers face high costs associated with health care and compensation for workers with occupational skin diseases, including retraining, and potentially decreased productivity (17). As such, there is growing interest in measurement of skin surface pH in non-clinical settings such as the workplace, as it may influence actual exposure to biologically active chemicals, as well as whether contaminants will penetrate into the underlying viable epidermis and become biologically active.

Guidelines exist for measurement of skin surface pH in highly controlled clinical settings (2); however, non-clinical settings may be more variable because of a number of factors. The workplace represents a ‘worst case’ environment for *in vivo* measurement of skin surface properties. In non-clinical settings, investigators may have less control over the measurement conditions (e.g. temperature, humidity, time of day, seasonality, time away from work, and skin contamination) or subject behavior (e.g. use of skin cleansers, barrier creams, and use of gloves or other occlusive garments). These factors may increase variability in data which can make interpretation challenging. Hence, there is a need for guidelines on non-clinical (e.g. workplace) measurement of skin surface pH to standardize data collection and reporting. In response to this need, an expert workshop was convened as part of the 5th International Conference on Occupational and Environmental Exposure of Skin to Chemicals (OEESC) held in Toronto, Canada in June 2011. This study presents a consensus summary of workshop participants for guidelines and best practices for measuring skin surface pH in non-clinical settings such as the workplace.

## Instrumentation and Measurement Principles

The universal method for measuring skin surface pH is the glass planar electrode connected to a voltage meter. Currently, there are four commercially available glass planar electrode instruments (2, 22):

- pH meter 1140 (Mettler-Toledo, Greisensee, Switzerland),
- Skin pH-meter 900 or 905 (Courage & Khazaka, Köln, Germany),
- Russell pH Ltd (Auchtermuchty, Fife, UK), and
- pH meter (Radiometer, Copenhagen, Denmark).

The skin surface is hydrophobic and does not contain pure liquid water. Rather, the surface film is an aqueous mixture containing lipids. When using an electrode to measure pH on the surface of the skin, amphiphilic-free fatty acid lipids release H^+^ ions into water applied to the skin by the electrode. In chemistry, pH = −log[H^+^]; however, skin liquids are not a pure aqueous solution. As such, this definition is not appropriate for skin pH measurement because H^+^ ions are not in a pure solution at the surface of skin. What is measured at the skin surface is referred to as *apparent skin pH* because it is unknown whether surface pH actually reflects H^+^ ion concentration of intracellular water or if it represents the combined acidity of corneocytes, lipids, and water-soluble compounds diffusing into water applied to the surface by the electrode (2, 22).

## Factors Influencing Skin Surface pH Measurement

Table 1 summarizes endogenous, exogenous, and environmental factors that may affect skin surface pH. In addition, experimental and instrumentation factors may influence measurements, although many of these factors can be controlled or minimized by using a well-developed protocol.

**Table 1:** Endogenous, exogenous, and environmental factors affecting skin surface pH

Factor	Ref	Sex	*N*	Ethnicity	Age (years)	Effect*
Endogenous						
Anatomical position	(29)	M, F	12	Caucasian	NR	+
	(26)	M, F	83	Korean	23–37	+
	(23)	NR	20	NR	24–83	+
	(27)	F	20	Japanese	22–37	+
	(24)	M, F	125	NR	21–57	+
	(28)	M, F	574	Caucasian	18–95	+
	(32)	M, F	22	Japanese	20–40	–
	(25)	M, F	200	NR	19–27	+
	(30)	F	33	NR	33†	–
Gender	(29)	M, F	11	Caucasian	NR	+
	(26)	M, F	83	Korean	21–37	+
	(28)	M, F	574	Caucasian	18–95	–
	(25)	M, F	200	NR	20–27	+
	(36)	M, F	28	NR	NR	–
	(33)	M, F	14	NR	25–49	+
	(37)	M, F	12	NR	24†	+
	(38)	M, F	443	Chinese	13–70	+
Age	(23)	NR	20	NR	24–83	+
	(28)	M, F	574	Caucasian	18–>80	+
	(33)	M, F	14	NR	27–71†	±
	(38)	M, F	713	Chinese	0.5–95	+
	(42)	F	500	NR	20–70	–
Ethnicity	(39)	F	30	Caucasian	18–45	±
		F	30	Black	18–45	±
	(40)	F	10	Caucasian	42†	–
		F	8	Black	42†	–
Skin health	(5)	NR	NR	NR	NR	±
	(6)	M	13	NR	21–71	+
	(7)	NR	NR	NR	NR	+
	(8)	NR	27	NR	NR	+
Rhythmicity/Circadian rhythm	(53)	M, F	16	NR	23–53	+
	(34)	F	80	Caucasian	21–34	+
	(35)	M, F	12	NR	NR	+
Exogenous						
Washing	(5)	NR	1	NR	NR	+
	(43)	M, F	10	NR	21–38	+
	(45)	M, F	10	NR	23–32	+
	(44)	M, F	120	NR	20–25	+
Occlusion	(46)	M, F	26	NR	21–60	+
	(47)	M, F	10	NR	NR	+
Environmental						
Seasonality	(48)	M, F	24	Japanese	19–55	+

M, male; F, female; *N*, number of subjects; NR, value not reported.

* Effect: + = has an influence; − = no influence; ± = inconclusive data

† Average value.

Endogenous factors of importance for workplace measurement of skin surface pH include anatomical position (e.g. wrist or face), skin health, and chronological rhythms. Values of skin surface pH vary between anatomical positions (e.g. forehead vs. forearm) (23–25) and among sites of a given position (e.g. cheek, forehead, and nose on the face) (24, 26–28). One study reported that pH on the flexor skin surface of the forearm of men was more acidic near the wrist than the elbow, although no difference was observed for women (29). Kleesz et al. (24) did not observe differences in skin surface pH between the forearm and elbow in a cohort of men and women. In one study, no difference in skin surface pH was observed between the dominant and non-dominant forearm (30). Data on right/left differences in skin pH on the hands and forearms are conflicting (24, 29). Some diseases may influence skin surface pH in adults. Loss-of-function mutations in the gene encoding filaggrin, a protein responsible for maintaining skin barrier integrity, is a significant factor that predisposes persons to atopic dermatitis (31). Many, but not all persons with atopic dermatitis have elevated skin surface pH (31, 32), which supports the premise that the increase is the result of fillagrin mutation status and not atopic dermatitis per se (8). For persons with ichthyosis (6) and irritant contact dermatitis (7), skin pH may be elevated compared with control subjects. One study reported circadian rhythmicity for skin surface pH with maximal values in the afternoon (between 14 : 00 and 16 : 00) and minimum values in the evening (at approx. 20 : 00) (53). Le Fur et al. (34) detected time-dependent changes for skin surface pH on the face but changes were not circadian; pH had a minimum value during the night (at approx 04 : 00). Ehlers et al. (35) reported that skin pH at various sites on the forearm decreased during the working day. Data are conflicting on the role of gender (25, 26, 28, 29, 33, 36–38) and ethnicity (39–41) in skin surface pH. Age-dependent differences in skin surface pH have been reported for people aged 70–95 years (23, 28, 36, 38, 42), which is generally outside of normal working years.

Exogenous factors of importance for measurement of skin surface pH in the workplace are hygiene practices (i.e. frequency of hand washing, use of soaps or detergents), use of topical products (i.e. lotions, barrier creams, and cosmetics) and occlusion. Alkaline soaps tend to raise skin surface pH (5, 43, 44) whereas acidic soaps tend to cause only a slight increase or even a lowering of skin surface pH (44, 45). The effects of soaps, synthetic detergents, and topical products may be transient with skin pH returning to baseline in as little as 90–120 min (1, 43, 44). Long-term (3–4 days) occlusion of skin increased skin surface pH and required about 1 day to return to baseline (46, 47). However, data are lacking on the short-term (hours) influence of occlusion on skin surface pH which is more relevant to workers who wear gloves or other chemical protective garments intermittently during their shift.

Abe et al. 1980 investigated seasonal changes over 1 year for young Japanese women and observed that skin pH was significantly lower in July but equal in January, April, and October. These results suggested that seasonality can influence skin surface pH, though the atmosphere of the room in which measurements were performed was not controlled. Although the influence of seasonality on skin surface pH remains unclear at this time, it may be pertinent for some workers, especially those employed outdoors.

## Measurement Protocol for Non-Clinical Settings

The purpose of this protocol is to provide guidelines for measurement of skin surface pH in non-clinical settings by accounting for, and where possible, minimizing the influences of endogenous (i.e. anatomical position, skin health, time of day), exogenous (i.e. topical products, occlusion), environmental (seasonality), and instrumentation factors. Prior to performing any measurements, information on the purpose(s) of the study, risks and benefits of participation, and any other pertinent information should be clearly communicated to each study participant. Informed consent must be obtained from each participant in accordance with the human subject policy of the institution(s) governing the study. Upon obtaining informed consent, precise instructions should be communicated to participants regarding hygiene practices (skin washing) and the use of topical products (cosmetics, lotions, etc.) acceptable for the study data collection goals. Appendix A in the companion study (49) is a checklist for use during workplace measurement of skin surface pH (as well as trans-epidermal water loss and/or SC hydration). Use of this checklist is intended to ensure collection of pertinent data relating to workplace measurement conditions that are critical for data interpretation and in turn will promote inter-study comparability.

### Preparation and handling of pH electrode and meter

The pH electrode should be handled according to the manufacturer's instructions and should never be brought into forceful contact with hard objects. It is important to ensure that the height of electrode fill solution in the external (reference) electrode sheath is higher than the level in the measuring electrode (4). Prior to use, the pH electrode and the meter should be equilibrated in the same environment in which measurements will be taken for at least 20 min (2).

At a minimum, the pH electrode must be calibrated using standard buffers prior to performing any measurements. Calibration should be performed in accordance with the electrode manufacturer's instructions; at a minimum, a two-point calibration is recommended. The pH level of calibration buffers should span the expected skin surface pH values, which may vary from 2 to 8 (50). Calibration should be performed at a frequency specified by the manufacturer, or daily, and verified periodically using a standard buffer.

### Measurement of skin surface pH

Prior to measurement of skin surface pH, a study participant should be acclimated to the measurement environment to avoid errors caused by temperature or sweating. The European Group on Efficacy Measurement of Cosmetics and Other Topical Products (EEMCO) recommend that for clinical studies a subject would ideally be acclimated for at least 20 min at an ambient temperature (20–22°C) and relative humidity (40–60%) (2). In non-clinical settings such as the workplace, it may not be feasible for a worker to leave their shift long enough to acclimate for 20 min plus time for measurements. Workers may be unwilling to have measurements performed on their own time, either before or after a shift, for personal reasons. In addition, the environmental conditions recommended by EEMCO may not be achievable in non-clinical settings. For example, depending upon the season, doors, and windows may be open (or closed) to cool (or heat) the workspace. In our experiences with occupational hygiene surveys, a facility manager may provide researchers with workspace where there is little control over the ambient temperature and humidity levels. We recommend that measurement conditions be controlled and characterized as far as reasonably practical such that obtained data will meet study goals. Avoid making measurements during conditions of extreme heat or cold.

As noted, exogenous factors such as washing, use of topical products, and occlusion may influence skin surface pH. For clinical studies, EEMCO recommends that skin surface pH measurements be made 2–3 h after washing with tap water, 5 h after washing with synthetic detergents, and 10 h after washing with alkaline soaps (2). Ideally, measurements should be made 12 h after use of ointments, body lotions, and other topical products in the intended measuring area (51). Performing measurements after such long lag times is generally not possible in the workplace, particularly among health care workers and food handlers who frequently wash their hands or cosmetologists who frequently handle lotions and topical products. We recommend making measurements in non-clinical settings before washing or application of ointments and lotions if feasible (e.g. prior to the start of a work shift and before the end of a shift). Finally, no measurements should be made in clinically inflamed skin or adjacent to such areas. If the desired measurement position is affected by a skin disease or injury, a disease- and injury-free position in close proximity may be used instead; however, caution is warranted as skin surface pH varies by anatomical position (Table 1). If a reasonable substitution for the desired position cannot be identified, it may be necessary to exclude the participant from the study. In workplace studies, acute changes in skin surface pH during normal working procedures are of interest. If measurements cannot be made before washing or application of topical products, the researcher is cautioned that use of alkaline soaps may raise skin surface pH (5, 43) whereas acidic soaps may lower skin surface pH (45) and such influences should be considered in data interpretation. Conformance with study protocol instructions for washing and use of topical products, the absence/presence of skin inflammation, and chosen anatomical position should be verified and any deviations noted before making measurements.

Some work tasks may require use of highly occlusive protective garments, such as nitrile or latex gloves and coverall suits, made of synthetic textiles. Clothing made of natural textiles (e.g. cotton) may also be occlusive in some work situations. As such, it is important to verify whether the volunteer wore protective garments over an anatomical position and record the temporal relationship (how long before skin pH measurement) and for how long the skin was occluded prior to pH measurement.

Measurement of skin surface pH should be made at anatomical positions appropriate for the study design and the workplace. For example, if in a particular facility workers do not wear a respirator then cheek or neck skin may not be occluded. However, study goals must also be considered and as noted above, values of skin surface pH vary among sites on the face (24, 26–28, 32), which may not make this location desirable. In clinical studies, the standard anatomical position for skin surface pH measurement is the (mid) volar forearm away from the wrist (4). Even if another anatomical position is of interest for a particular study design, it is recommended to measure skin surface pH at the mid volar forearm as a standard measure to put results into perspective.

Care should be taken that no cosmetic residue or excess sebum is on the skin surface at the measurement location (2). If necessary, the skin can be wiped using a clean, dry, oil- and lotion-free substrate, such as tissue paper (e.g. Kimwipe®, Kimberly-Clark, Roswell, GA, USA) or filter paper (e.g. Whatman ashless circles), although use of water is not recommend as it can affect skin surface pH values. The electrode surface should be moistened using distilled water prior to placing it on the skin. As recommended by EEMCO, a standard volume of 20 μL distilled water should be applied to the skin and the electrode placed on the water to ensure comparability of measurements (2). The pH electrode should be held at a right angle to the skin with gentle pressure to ensure optimal contact (29, 35); avoid applying excessive pressure which can affect the volume of liquid at the interface of the electrode and skin (2). Measurements should be recorded when a stable signal (as defined by the instrument manufacturer) is achieved.

The number of measurements, anatomical position (e.g. forearm), and anatomical site (e.g. midway between the wrist and elbow) are important considerations. If simultaneous assessment of skin exposure to a contaminant is also a study goal, the area of pH measurement should be as close as possible to the area monitored for skin exposure without confounding the respective measurements. In published clinical studies, the number of measurements per anatomical position ranged from two (30, 33, 34) to four (47). We recommend that three sequential measurements be made at the same anatomical position, with a consistent and reasonable time lag between measurements, and the results -averaged. The choice of lag time between measurements cannot be too long, especially if performed during a work shift. We recommend 5 s between measurements, although this interval may not be appropriate for all studies, in which case it is emphasized that the lag time used should be as short as feasible and applied consistently. To reduce contamination of the electrode, Ehlers et al. (29) recommend rinsing it with deionized water after every three measurements. To avoid any possible effect of electrode contamination on skin pH measurements, the user may wish to rinse it prior to each measurement. Furthermore, it is recommended that all measurements be made at a given anatomical position before moving to the next position. If repeat measures of skin surface pH will be made at a given anatomical position (e.g. pre- and post-work shift), record both sets of measurements at the same position to reduce error. One can ensure that the same anatomical position is measured each time by photographing the measurement location and/or using a template. In our own experiences, measurements obtained by different researchers may introduce a source of variability; therefore, we recommend that the same person perform all measurements. While modern clinical studies may report skin pH values with precision of ± 0.01 units, for workplace studies we recommend a precision of ± 0.1 units. Between measurements, the electrode and meter should be stored in accordance with the manufacturer's instructions.

### Interpretation of skin surface pH measurements

As summarized in Table 1, values of skin surface pH may vary as a result of a number of factors. In addition, consensus is lacking with regard to reference values for skin surface pH (normal or diseased skin). Hence, comparison of absolute values of skin surface pH within and between research studies is problematic. As such, we recommend that results for a given anatomical position be reported and compared as a relative (or percent) change in pH values. For example, if the aim of a study is to assess acute changes in barrier function resulting from exposure and/or workplace conditions, then quantifying the difference in skin surface pH relative to baseline (prior to start of shift) for a worker would be appropriate. If the aim of a study is to assess chronic changes caused by disease, then expressing the difference in skin surface pH between a worker and control subject as a percentage is preferred over absolute values.

Skin surface pH results at a given anatomical position should be expressed with a measure of central tendency (i.e. arithmetic mean or median value) and variability (i.e. standard deviation or percentiles). Note that regional differences in skin pH (e.g. on the face) may be the result of variations in sebum content (7) and thus one cannot directly compare skin surface pH at sebum-rich sites to sebum-poor sites (2). As such, it may be useful to measure skin sebum content (e.g. using a Sebumeter®, Courage & Khazaka, Köln, Germany) at the beginning and end of a shift and adjust for sebum secretion during skin pH data analysis. If the aim of a study warrants use of a control group, the volunteers should be matched to workers with respect to relevant endogenous, exogenous, and environmental factors and measurements made in a similar environment with the same instrumentation to ensure consistency in data collection.

## Data Reporting

A minimum data set should be reported with study results. The rationale for the importance of the relevant endogenous, exogenous, environmental, and experimental factors is described in the preceding text and in Table 1. The following minimum information must be reported:

- Endogenous factors
  - The anatomical position(s) and exact site(s) of skin surface pH measurements and a rationale for the choice of site(s). The choice of anatomical position and sites will be specific to the study design of the research.
  - Skin health at time of surface pH measurements. For measurements on hands and wrists, this can be documented and assessed using, for example, a validated teledermatologic toolkit for standardized hand photographs in non-clinical settings. In one study (52), the intra-rater reliability of this tool showed a high agreement between direct visual inspection and the photographic assessment with a positive likelihood ratio of 7.4 and a negative likelihood ratio of 0.07 (88% complete agreement, kappa 0.79). Furthermore, the inclusion of a skin symptoms questionnaire for current symptoms might be advisable.
  - Date and time of day when skin surface pH measurements were performed. Note that if skin surface pH is to be quantified on different days for the same employee, to the extent feasible, measurements should be made at the same time of day to minimize any time-dependent effects (34, 53).

- Exogenous factors (see also questionnaire in appendix of companion paper)
  - Hygiene practices (washing) prior to measurement, including conformance or deviations from instructions given to study participants.
  - Use of any topical products, including conformance or deviations from instructions given to study participants. Note also if skin was dry wiped before measurement because of the use of topical products in the preceding 12 h.
  - Use of any protective garments or other materials that might have caused occlusion of the skin, including the type of covering, frequency and duration of use, and time since last use.

- Environmental factors
  - Season during which skin surface pH measurements were made, including typical average outdoor temperature and humidity levels for the geographic region.

- Experimental and instrumentation factors
  - Equilibration time of pH meter in measurement environment during study.
  - Calibration of pH meter and electrode, including pH values of standard buffers used for the study.
  - Frequency with which the pH meter and electrode calibration was verified during the study.
  - Acclimatization conditions in room where measurements were made during study, including duration spent by study participants and temperature and relative humidity of atmosphere.
  - Workplace temperature and humidity, especially if employee works in an atmosphere that is significantly different (e.g. furnace room) from the acclimatization atmosphere.
  - How the pH electrode was applied to the skin surface, including time to achieve a stable measurement in accordance with the manufacturer instructions during study.
  - The number of measurements per anatomical position, lag time between measurements, whether repeated measurement were made adjacent to one another or in exactly the same position for each worker.

- Measurement interpretation
  - Results reported (and compared) as a relative (or percent) change rather than absolute change in values.
  - Results expressed as a measure of central tendency (arithmetic mean or median value) and variability (standard deviation or percentiles).

- Any notable deviations from the guidelines in this protocol.

## Summary

Skin surface pH is one factor that affects contaminant dissolution and/or partitioning, which in turn influences permeation into the underlying viable epidermis. Exposure to chemicals that reach the viable epidermis may result in skin diseases, such as allergic or irritant dermatitis, which may place a considerable financial strain on both the employee and employer alike. To avoid the burden of occupational skin diseases, efforts must be made to reduce and prevent exposures. As such, there is an emerging perspective that it is not sufficient to only assess exposure to an agent of concern; it is also important to understand the condition of the skin at the time of exposure. We presented a consensus summary of guidelines and best practices for measurement of skin surface pH that is broadly applicable to non-clinical settings, with emphasis on the workplace. Key points of these guidelines are: (i) minimize, to the extent feasible, the influences of endogenous (i.e. anatomical position, skin health, time of day), exogenous (i.e. topical products and occlusion), environmental (seasonality), and measurement (atmospheric conditions) factors; (ii) report results of skin surface pH measurements as a difference or percent change (rather than absolute values) using a measure of central tendency and variability (i.e. arithmetic mean and standard deviation); and (iii) accurately report notable deviations from these guidelines and all factors listed in the data reporting checklist. It is our intention that these guidelines provide consistency in non-clinical measurement and reporting of skin surface pH data which is essential for inter-comparison of study results.
